# Digital measurement of anterolateral knee laxity using strain sensors

**DOI:** 10.1007/s00402-023-05024-4

**Published:** 2023-08-17

**Authors:** Hermann O. Mayr, Nikolaus Rosenstiel, Karthika S. Prakash, Laura M. Comella, Peter Woias, Hagen Schmal, Michael Seidenstuecker

**Affiliations:** 1 Department of Orthopedic and Trauma Surgery, Faculty of Medicine, Medical Center-University of Freiburg, Hugstetter Straße 55, 79106 Freiburg, Germany; 2 Department of Microsystems Engineering IMTEK, Albert-Ludwigs-University of Freiburg, Georges-Koehler-Allee 103, 79110 Freiburg, Germany; 3 G.E.R.N. Center of Tissue Replacement, Regeneration & Neogenesis, Department of Orthopedics and Trauma Surgery, Faculty of Medicine, Medical Center-University of Freiburg, Albert-Ludwigs-University of Freiburg, Hugstetter Straße 55, 79106 Freiburg, Germany

**Keywords:** Rotational laxity knee, Digital measurement, Strain sensors, Measurement knee rotation, Anterolateral knee laxity

## Abstract

**Purpose:**

The ambition of the research group was to develop a sensor-based system that allowed the transfer of results with strain sensors applied to the knee joint. This system was to be validated in comparison to the current static mechanical measurement system. For this purpose, the internal rotation laxity of the knee joint was measured, as it is relevant for anterolateral knee laxity and anterior cruciate ligament (ACL) injury.

**Methods:**

This is a noninvasive measurement method using strain sensors which are applied to the skin in the course of the anterolateral ligament. The subjects were placed in supine position. First the left and then the right leg were clinically examined sequentially and documented by means of an examination form. 11 subjects aged 21 to 45 years, 5 women and 6 men were examined. Internal rotation of the lower leg was performed with a torque of 2 Nm at a knee flexion angle of 30°.

**Results:**

Comparison of correlation between length change and internal knee rotation angle showed a strong positive correlation (*r* = 1, *p* < 0.01). Whereas females showed a significant higher laxity vs. males (*p* = 0.003).

**Conclusions:**

The present study showed that the capacitive strain sensors can be used for reproducible measurement of anterolateral knee laxity. In contrast to the previous static systems, a dynamic measurement will be possible by this method in the future.

## Introduction

The differentiated diagnosis of rotational laxity of the knee joint is the decisive prerequisite for the correct therapeutic procedure [1]. Until now, the detection of knee laxity has mostly been based on the subjective clinical tests that depend on the examiner. Rotational laxity of the knee joint with anterior cruciate ligament rupture is clinically assessed by the pivot shift test [2]. Because the examination depends on the technique of the examiner, a semi-subjective result is achieved. The commonly used commercially available arthrometers KT 2000 and Rolimeter [3] are able to objectively measure anterior − posterior translation, but the rotational component is not taken into consideration. Moreover, the results can vary depending on the patient's position, the examiner's dominant arm, and involuntary muscle contractions [4]. Even the latest measuring devices such as the "Automatic Knee Arthrometer (AKA)" only measure anterior − posterior translation [5]. The Telos device [6] can only verify varus/valgus laxity translation and anterior − posterior translations. While electromagnetic sensors using the Robotic Knee Test (RKT) System can reliably determine knee rotation, it is a static system with considerable technical effort [7]. According to the clinical experience, the applied high torque often leads to muscular counterstrain of the patient and pain. Moreover, general anesthesia is required for some of these measurements [8]. Because of the complexity, they are rarely applicable in routine clinical diagnosis. Computer navigation [9–14] is useful only in the context of surgical procedures. Although it provides reliable results, it is more useful for validation of surgical techniques.

Cadaver studies using amputates [15–17] are important for exploring the basics. However, the biomechanical properties of the tissue are altered in the cadaver, so that transferability of the results to in vivo situation is limited. In some cases, clinically intolerable high forces are applied to the knee joint. The force is often transmitted directly through the bone, creating conditions that cannot be reproduced in everyday clinical practice. Nevertheless, these studies are trend setting and important in basic research. Devices from the early days of rotation measurements such as the "Rottometer" [18] showed a high difference between the suggested vs. actual lower leg rotation. Owing to the low frictional connection between the lower leg, ankle joint and foot on the one hand, and the receiving fixation mechanism on the other hand, this bias is favored. In the "rigid" boot of the working group of Tsai [19], this problem was already partially taken into account. Nevertheless, when the knee was in a neutral position, a considerable rotational capacity remained in the joint. Quinn et al. [20] already described in 1991 for the axial examination in internal and external rotation that significantly higher three-dimensional forces and torsional moments act on the knee joint during rotational fixation of the foot and ankle. A validated device developed by the current research group to measure rotational laxity [21–25] takes this fact into account by fixing the talus in a defined dorsal extension and locking the ankle fork. The advantage of the device described here is the possibility of a controlled measurement of the rotation of the lower leg at a defined torque. In addition, it is a purely mechanical and easily transportable device. The sensitivity of most commercially available arthrometers must currently be seen as a weakness in the experimental setup. However, even the current rotational laxity measurement device is a stationary device and thus does not allow laxity measurement under daily physical activity. Therefore, it is the ambition of the research group to develop a sensor-based system that allows the transmission of the results with stretch marks applied to the knee joint and thus also allows mobility of the patient [26]. In the present study, this system was validated in comparison to the current static mechanical measurement system. For validation, the internal rotational laxity of the knee joint was measured, as it is relevant for injuries of the anterolateral ligament complex of the knee and the ACL [27, 28].

Hypothesis: Capacitive strain sensors can be used for reproducible laxity measurement of the anterolateral knee laxity.

## Materials and methods

### Measuring system

The measuring system consisted of a capacitive strain sensor connected to a flexible circuit board. The electronics on the circuit board were used for data acquisition and transmission. When the sensor strip is stretched, the distance between the fingers of the strain sensor changes, hence the capacitance changes. Consequently, the strain can be measured via the change in capacitance. The measurement system was taped to the knee, with the sensor attached to the skin in the direction of the anterolateral ligament (ALL) at a knee flexion angle of 30°. The bottom of the sensor (made of silicone) and the bottom of the circuit board (made of FR4), was in contact with the knee surface. Importantly, the sensor was attached to the knee by a crosslinking silicone rubber without prestretching. The crosslinking silicone rubber is an orthopedic product. The measuring system was designed to maintain full functionalities for two full days of examination but in the present study, 7 patients were measured for 90 min for one day. During a patient session the knee rotation angle was recorded and monitored by the sensor extension. In the present study, the change in length of the capacitive strain sensor and the associated change in capacitance, expressed by the capacitance charge times, was measured by internal rotation of the knee joint with a defined torque. Capacitance reading was performed using Texas Instruments CC2652R1 ultra-low-power microcontroller. The capacitance was measured using a TDC (Time to digital converter) embedded in the chip. The working principle of the TDC is to measure the charging time of the capacitor which is proportional to the capacitance value [29]. The sensed value is therefore the capacitance charge time, which is proportional to the sensor elongation. The sensor system was used under the constant supervision of physicians specialized in knee surgery and who are familiar with the use of the device. The reliability of the measurement method was defined as primary endpoint.

### Measurements

**Fig. 1:**
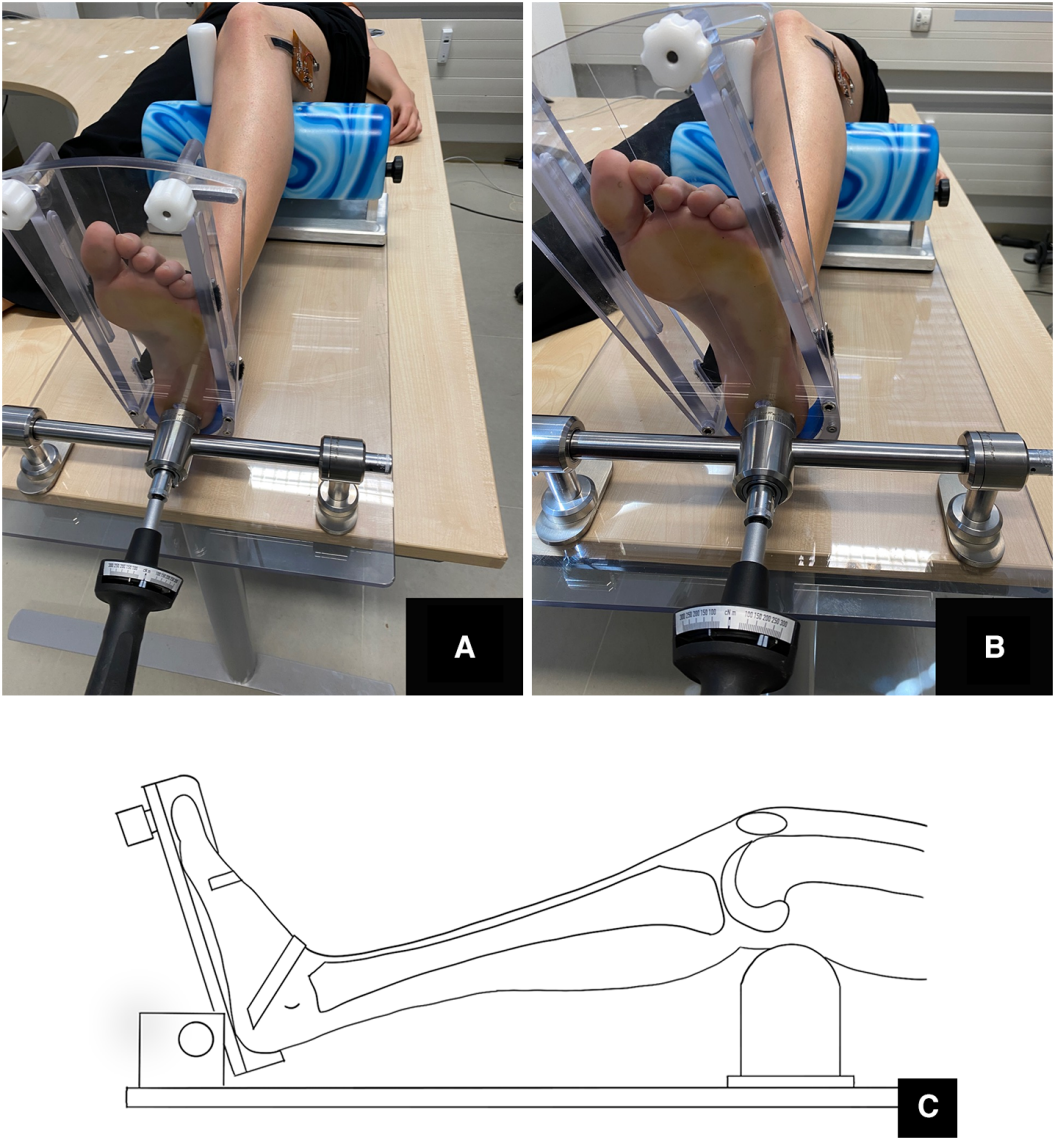
**A** Longitudinal positioning of the test person on the couch; **B** Medial support of the knee and lateral positioning of the capacitive strain sensor; **C** Alignment of the ankle and knee

The subjects were placed in supine position. First the left and then the right leg were clinically examined sequentially and documented by means of an examination form (see attached document). Exclusion criteria were the occurrence of pathological findings of ligamentous pathology in the area of the collateral/cruciate ligaments, previous knee operations or restrictions of the range of motion. There were 11 subjects aged 21 to 45 years, 5 females and 6 males. Subjectively, there were no problems in the knee area in any of the subjects. The Laxitester (Ortema Sports Protection, Markgroeningen, Germany) was validated for quantitative measurement of rotational laxity in the knee joint. This has been performed for ligament stable and unstable knees [23, 24]. Because of the large interindividual differences, a comparison was made between the left and right knee. The subject was then placed in supine position on the Laxitester with the respective leg to be examined in the body axis (Fig. 1). Previously, a calibration was performed to determine the length change of the sensor via the capacitance change.

The knee circumference was measured for each subject. The length change of the strain sensors was corrected according to the knee circumference in order to obtain comparable nondimensional values. To do this, the length change of the respective strain sensor was divided by the circumference of the knee joint over the middle of the patella.

The study was approved by the responsible ethics committee (approval number 487/16).

**Fig. 2:**
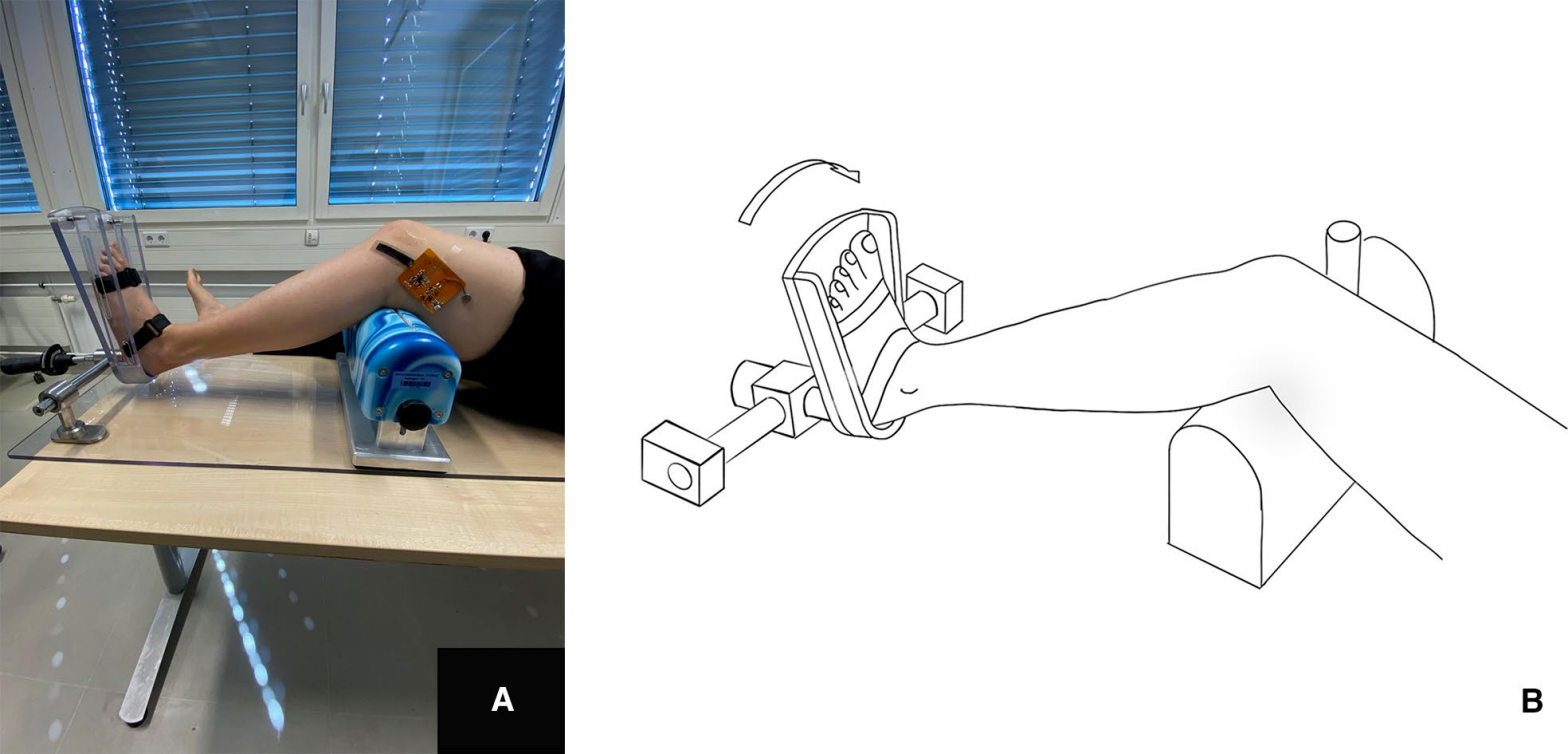
Positioning of the strain sensor in the course of the ALL and internal rotation of the lower leg with a torque of 2 Nm; the strain sensor was glued in the course of the anterolateral ligament below the lateral epicondyle of the femur to Gerdy’s tubercule

The distal femur rested on a support bench with which a knee joint flexion of 30° was set by height adjustment. The femoral condyles were secured against rotation in the positioning aid by means of a medial post. This also prevented internal rotation and displacement of the femur. The foot was strapped into the holding device without footwear. The second beam was centrally aligned via an adjustable side clamp on the medial and lateral foot edge. In accordance with the above-mentioned previous studies, dorsiflexion was set to 2 Nm using a torquemeter in order to exclude rotational laxity in the ankle joint. The strain sensor was glued in the course of the anterolateral ligament below the lateral epicondyle of the femur to Gerdy’s tubercule. Internal rotation of the lower leg was performed with a torque of 2 Nm at a knee flexion of 30° (Fig. 2).

### Statistics

Descriptive statistics, linear regression and Spearman's rank correlation were performed. Statistical analysis was performed using SPSS software, version 12.0 (SPSS Inc., Chicago, IL, USA). Basic descriptive statistics were used to describe the patient population. Group differences were tested by age and sex, and measured length change were compared between participants at different rotation angles in 5° increments (10°, 15°, 20°, 25°, 30°, 35°, 40°). Scatter plots were used to evaluate the differences between participants’ values and the variations between different angles. The relationship between the length change and the angle of knee rotation was measured using Spearman's rank correlation to evaluate the monotonic relationship between two variables in non-normal distributed data. The correlation between the change in length and the degree of knee rotation was measured for each side using Spearman's rank correlation coefficient (*r*) and significance level (*p*) for all participants, and the collected correlation data of different angles were plotted. Significance was set at 0.05. With a significance level of 5% for two-tailed testing, a power of 80% and an expected difference of 5° with a standard deviation of 4°, 11 subjects were required to obtain a statistically significant difference.

## Results

The current study included a total of 11 subjects of which 5 were female and 6 were male. The average age was 28 ± 6 years. Owing to the ethics committee vote, 11 subjects were allowed to participate during the Covid pandemic.

The results obtained from a simplified knee joint model exhibited a reproducible relative capacitance change of 0.01 F for every 5° angle change from 10° through 45° rotation [30].

**Fig. 3:**
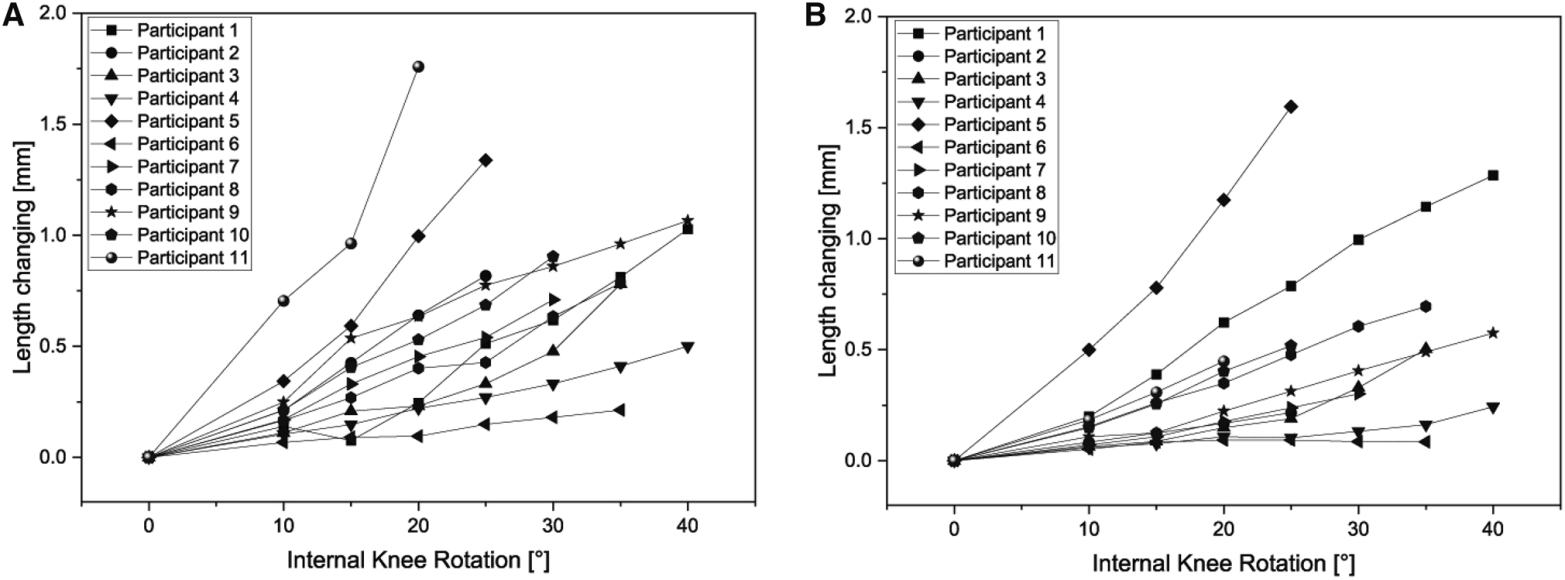
Comparison of correlation between length change and internal knee rotation for all participants; the measurements are individualized for each participant for left (**A**) and right (**B**) knee. Significances, please see Table 1. All measurements were performed at 30° knee flexion

**Fig. 4:**
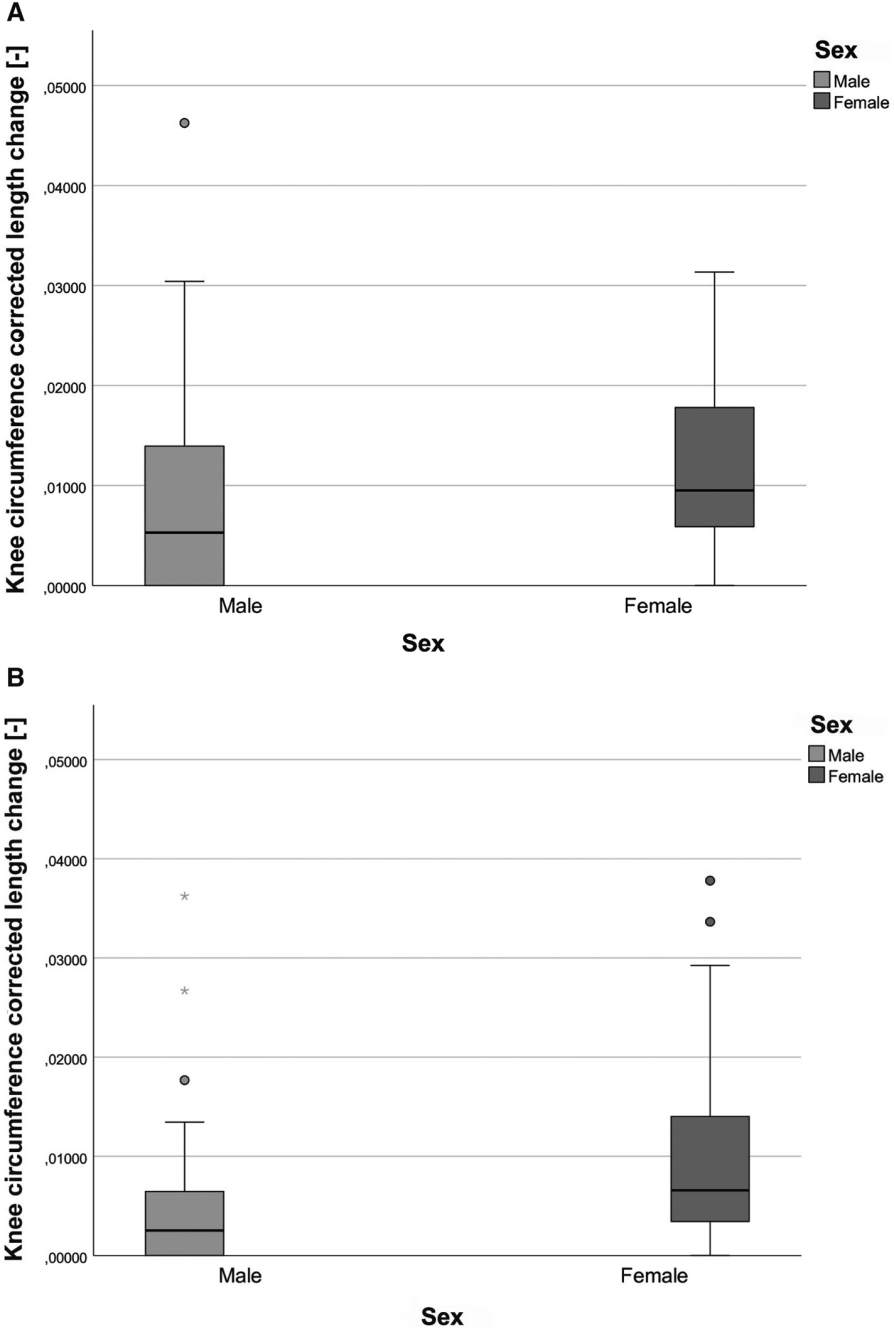

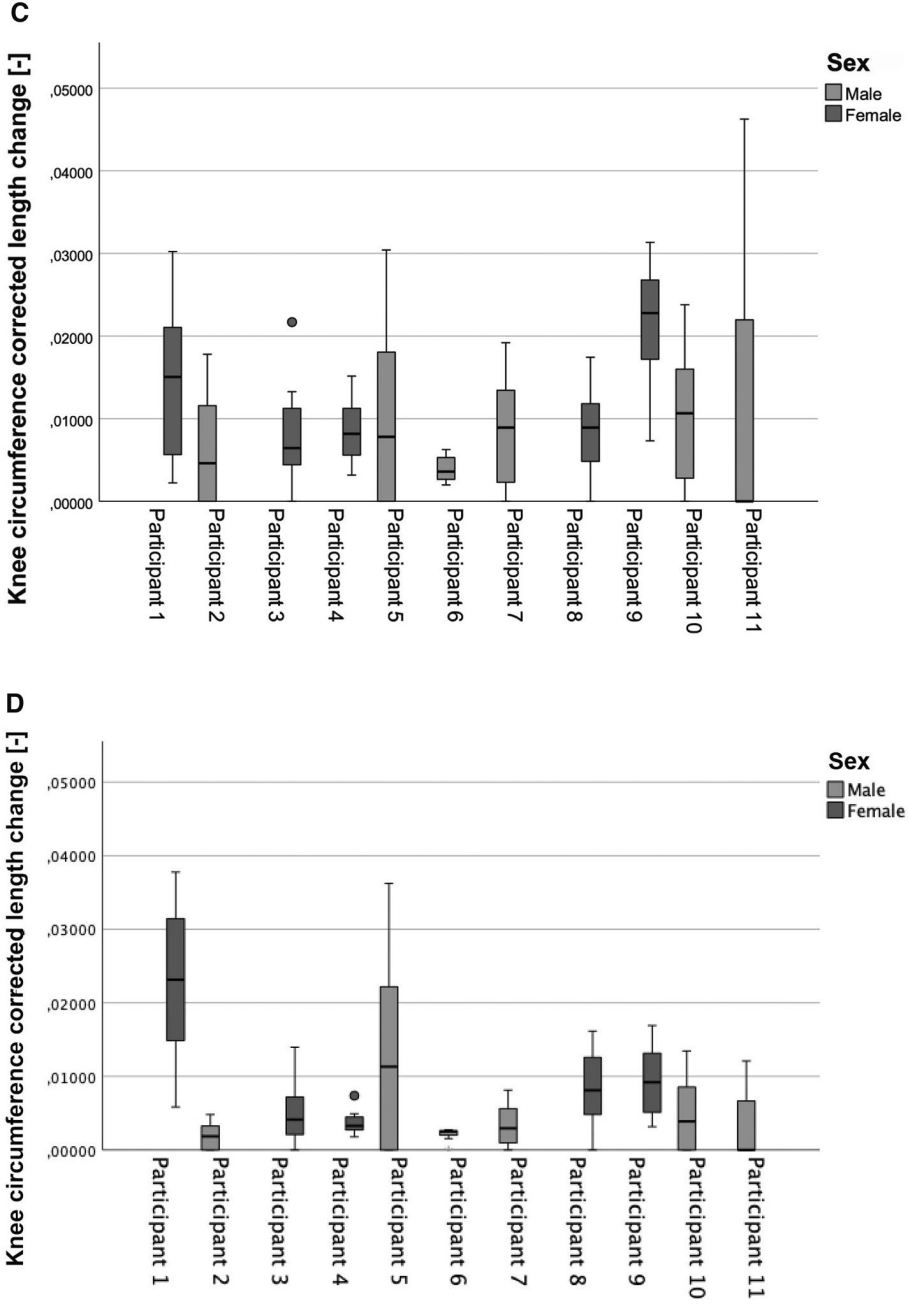
Box plots comparing the sensor length change on both sides; **A**, **C** = left knee; **B**, **D** = right knee, Top: differences between sex as groups; Bottom: differences between each participant; Male participants showed more variability in comparison with female participants. All measurements were performed at 30° knee flexion

A significant sex difference was noted in the range of internal rotation of the knee (*p* = 0.003). The range of rotation was larger in the female participants than in the male participants. As for the measured length changing values, the measurements presented showed an individualized result for each participant, which tended to decrease with increasing internal rotation angle, with no age or sex differences (Figs. 3 and 4). The stiffness of the strain sensor was in the linear range for all measurements [29] and within the first 20% strain of the sensor [30]. Thus, the differences determined do not result from a different strain of the sensor, but from individual differences of the test persons.

**Table 1:** Summary of linear regression analysis of association of sensor length change and angles of knee internal rotation (male participants are highlighted in light grey)

Participants/Side	Internal rotation angle [°] tibia	Length change [mm]	Beta coefficient	R^2^	p value	Inference
1 Left	35	1.03	0.955	0.911	< 0.001	Significant
1 Right	35	1.29	1	0.999	< 0.001	Significant
2 Left	40	0.82	0.988	0.977	= 0.001	Significant
2 Right	40	0.22	1	0.999	< 0.001	Significant
3 Left	30	0.78	0.936	0.876	= 0.002	Significant
3 Right	30	0.50	0.927	0.858	= 0.003	Significant
4 Left	40	0.50	0.994	0.989	< 0.001	Significant
4 Right	40	0.24	0.961	0.923	< 0.001	Significant
5 Left	40	1.34	0.981	0.963	= 0.003	Significant
5 Right	40	1.59	0.991	0.983	< 0.001	Significant
6 Left	35	0.21	0.990	0.975	< 0.001	Significant
6 Right	35	0.09	0.818	0.669	= 0.025	Significant
7 Left	25	0.71	0.994	0.989	< 0.001	Significant
7 Right	25	0.31	0.989	0.977	< 0.001	Significant
8 Left	25	0.78	0.986	0.972	< 0.001	Significant
8 Right	25	0.69	0.996	0.992	< 0.001	Significant
9 Left	30	0.45	0.987	0.975	< 0.001	Significant
9 Right	25	0.57	0.988	0.976	< 0.001	Significant
10 Left	20	0.90	0.993	0.987	< 0.001	Significant
10 Right	20	0.52	0.990	0.980	= 0.001	Significant
11 Left	35	1.76	0.974	0.923	= 0.026	Significant
11 Right	35	1.60	0.994	0.989	= 0.006	Significant

### Correlations between length change of the sensor and degrees of internal knee rotation

**Fig. 5:**
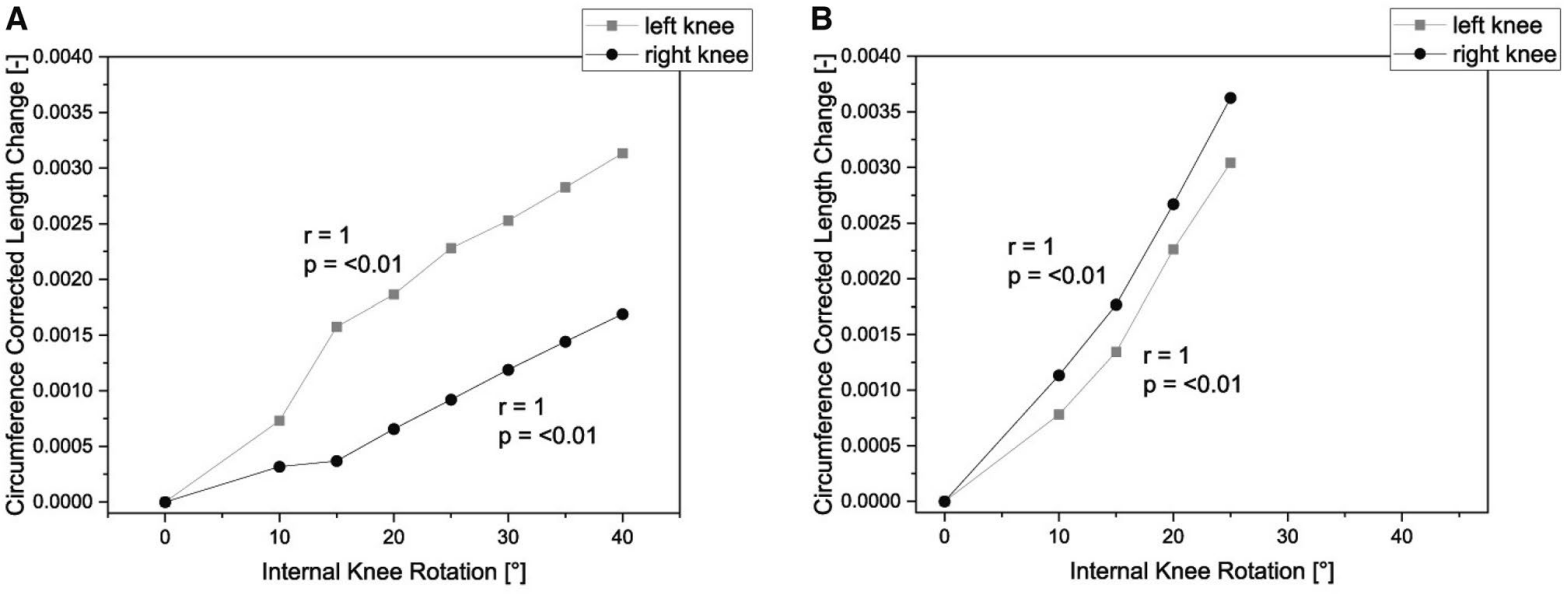
Comparison of correlation between length change and internal knee rotation angle for a single representative female (**A**) and male (**B**) participant. Whereas females showed a higher laxity. Spearman’s correlation coefficient r and significance level p are positioned in the graphs

Figure 5 shows an example of the correlations between the change in length (normalized to the knee circumference) and the degree of internal knee rotation for a female and a male subject. In 30° of knee flexion and a torque of 2 Nm female subjects were able to rotate the tibia internally up to 40° and male subjects up to 20°. In female subjects a significant difference between the right and left knee was seen; in male subjects, the difference was not as pronounced.

### Spearman's rank correlation

Spearman's rank correlation evaluated the relation of sensor length change and angles of knee internal rotation. The dependent variable length change was regressed on predicting variable knee internal rotation angles and it has significantly predicted length change in all the participants on both sides. Table 1 shows the summary of findings and the reliability. It is evident that some subjects showed hardly any difference in the change in length of the sensor with maximum internal tibial rotation of the knee between the left and right side, while others showed clear differences. This applied to both female and male subjects.

## Discussion

With the current work, it was possible to successfully evaluate for the first time a digital novel measurement method of anterolateral rotational laxity of the knee joint in vivo using capacitive strain sensors. The digital device was validated against a proven mechanical measurement system. The achieved precision and adaptability of the sensors showed a highly reliable applicability regarding the length change of the antero-lateral tissue strain for measurement of internal rotational laxity. In this content the importance of structures like the ALL, the anterolateral capsule, the iliotibial band or Kaplan fibers are documented in the recent studies [18, 31–33].

The measurement method of the strain sensor was established in vivo especially in the context of invasive blood pressure measurement, but has also been investigated on the cadaveric knee of humans [34, 35]. This measurement method using strain sensors which are applied to the skin in the course of the anterolateral ligament is based on the in vitro measurements on a simplified knee joint model of Zens et al. and on cadaveric knees of humans [26, 30]. Already in this in vitro experimental setup it could be shown that the bone-to-bone displacement was transferred to the skin and was measured there with the strain sensors. This could also be performed on human cadavers with capacitive strain sensors and KT-2000.

Owing to the Covid19 pandemic, the associated isolation, protective measures, the ethics committee to only allowed only 11 subjects. In order to avoid exposure risks, it was just possible to recruit members of the local university staff for the experiments according to the requirements of the ethics committee. To include patients with cruciate ligament pathologies was not approved by the ethics committee. Therefore, a new power calculation was necessary and the reliability of the measurement method was defined as primary endpoint.

The role of rotational laxity and subsequently extraarticular stabilizers was noted as early as 1989 at a meeting of the American Orthopaedic Society for Sports Medicine (AOSSM) at an expert conference [36]. In a current review Garcia-Mansilla et al. noted the importance of the anterolateral complex [27]. And especially in the case of recurrent anterior cruciate ligament ruptures, excessive rotational laxity is evident [21, 37]. The anterolateral soft tissue complex including the capsule, the Kaplan fibers and the IT band as a secondary stabilizer to the anterior cruciate ligament is increasingly understood [38, 39]. Some studies support the importance of the anterolateral ligament in anterior cruciate ligament reconstruction [40, 41].

In the current study, a significant sex-specific difference in internal rotational laxity of the knee joint was found. Previous studies examining hormone activity confirm differing ligament laxity between women and men. Sex differences in the biomechanical properties of tendons and ligaments are significant in the period of life in which the hormonal profile between women and men is significantly different [42].

Similar to the present results, a 2019 meta-analysis, including 76 studies, showed a side-to-side difference in ligament laxity of the knee joint. The asymmetry in laxity between internal and external rotation was significant for all flexion angles, with the largest mean difference at 30°. However, the dominant leg was not considered in this study [43]. By attaching the current strain sensors to the skin in direction of the anterolateral ligament and measuring the strain on the surface of the knee a dynamic and especially real-time measurement of the rotational laxity by a highly elastic strain sensor can be performed for the first time, unlike previous measurement methods (e.g. KT-2000 arthrometer, Rolimeter). Sex-specific studies of knee laxity are predescribed and were also shown in the context of other studies with a significantly higher laxity in women compared to men [44, 45] Thanks to the sensitivity of the sensor, we were able to detect for the first time a higher laxity of the dominant leg when comparing intraindividual differences [46]. Unlike static measurements on the model, however, a dynamic measurement method was used in vivo for the first time in the experimental setup and it was possible to show that the results are comparable to established measurement methods such as the Laxitester and, at the same time significant additional information could be gained [23]. Since the strain sensors are measuring a change in length and not a change in angle, a correction factor for the respective leg circumference was required for the conversion into an angle amount. During the measurements, it was noticed that there was often a significant difference in circumference between the two legs. Despite that, the reliability of the measurements was highly significant as shown in Table 1.

The next objective after the successful establishment of the strain sensors for the measurement of rotational laxity should be the use of the dynamic measurement system during activity or in everyday life. Up to now, investigations during rapid changes of direction, stair climbing or one-legged-hopping could only be carried out in the form of extremely time-consuming and error-prone motion tracking measurements on subjects in the laboratory due to a lack of other technical options [47]. The use of a strain sensor that is easy to produce is a unique argument for this application. Here, by integrating the strain sensor into a commercially available knee joint support a completely new possibility could be created to compare the patient's subjective impression of instability during activities such as walking upstairs or the one-legged hop with the objective measured laxity values through the easy-to-use strain sensor system. The possibilities may not be limited to the knee joint, but may also be further developed to examine ligament laxities at the elbow or ankle joint.

## Limitations

Due to the ethical regulations during the Corona Pandemic, a relatively large angle difference of 5° was specified for the power calculation. Patients with cruciate ligament pathologies were not included due to the ethical regulations.

## Conclusion

The present study shows that capacitive strain sensors can be used for reproducible laxity measurement of the soft tissue complex on the knee. In contrast to previous static systems, a dynamic measurement will be possible by this method in the future.

## Data Availability

The datasets generated and analyzed during the current study are available from the corresponding author on reasonable request.
